# Accounting for climate transition risk in banks’ capital requirements^☆^

**DOI:** 10.1016/j.jfs.2024.101269

**Published:** 2024-08

**Authors:** Lucia Alessi, Erica Francesca Di Girolamo, Andrea Pagano, Marco Petracco Giudici

**Affiliations:** European Commission - Joint Research Centre, Italy

**Keywords:** Climate transition risk, Climate stress-testing, Dynamic balance sheet, Banking crisis

## Abstract

This paper uses a stylized simulation model to assess the potential impact of climate transition risk on banks’ balance sheets in a climate-stress-testing (i.e. short-run) framework. We show that a moderate to high transition risk increases overall bank losses only relatively modestly if the baseline is a stressed macroeconomic scenario. However, even in a benign macroeconomic scenario, if high-carbon assets are at least 13% riskier than comparable assets a fire sale mechanism could amplify an initially contained shock into a systemic crisis, resulting in significant losses for the EU banking sector. We show that transition risks are concentrated, and find that an additional capital buffer of 0.9% risk-weighted assets on average would be sufficient to protect the system.

## 1 Introduction

Climate change, among its many other potential consequences, represents a new source of systemic financial risk, since its impacts have the potential to spread across the entire financial sector (see, for example, Bank for International Settlements, 2021a; Bank for International Settlements, 2021b; European Central Bank, 2021; European Systemic Risk Board, 2021). In particular, the literature groups climate-related financial risks into two macro-categories: (i) physical risks, which arise from catastrophic events becoming more frequent and more severe; and (ii) transition risks, which arise from the transition to a low-carbon economy and the related changes in strategies, policies and investments. The ability of financial institutions to identify in a timely manner and manage both types of risks, and to absorb financial losses potentially arising from them, is ultimately key for financial stability. Looking at the banking sector in particular, individual banks are exposed to climate-related risks to a varying degree, justifying a micro-supervisory approach. Against this background, this paper provides quantitative evidence on the magnitude of climate transition risk for the European banking sector by testing various scenarios, including that of a fire sale of high-carbon assets. We investigate under which conditions even a very small depreciation of such assets could trigger uncontrolled market dynamics, ultimately leading to significant losses for the banking system as a whole. We also show that the risks are concentrated in some countries. Based on these results, we propose a calibration for relevant prudential tools.

The idea of using prudential tools to address climate-related financial risks has received growing attention in the literature in recent years. Among others, Campiglio (2016) provides an early discussion about the use of macroprudential regulation to reward banks financing low-carbon activities. The paper analyses the role of differentiated reserve ratio requirements and capital requirements. D’Orazio and Popoyan (2019) provide a detailed overview of the available macroprudential tools, which could play an important role in leading the transition to a green economy. The paper focuses on, among other things, the role of capital requirements.

However, the quantitative impacts and the effectiveness of introducing differentiated capital requirements to support financing to specific sectors of the economy are unclear. For example, one such measure was introduced in 2013 in the EU, namely a capital discount for exposures to small and medium-sized enterprises (SMEs), also known as a ‘SME supporting factor’. An assessment by the European Banking Authority (2016) found no evidence that this factor increased access to finance for smaller firms relative to larger firms. In contrast, a study by Dietsch et al. (2021) shows that the SME supporting factor had a positive impact on credit supply in France.

Recent studies have investigated the effect of introducing a ‘green supporting factor’ (GSF) or a ‘dirty penalizing factor’ (DPF) to discourage financing of environmentally harmful activities. These supporting and penalizing factors are modelled assuming a range of variation of 15%–25%, such as the one used for the SME supporting factor (EU capital requirement regulation 2 allows a reduction in capital charges of up to 23.81% for investments below EUR 2.5 million and up to 15% for larger exposures). Dafermos and Nikolaidi (2021) examine the effect of a GSF or DPF that decreases and increases, respectively, the risk weight on loans by 25 percentage points. The authors conclude that green differentiated capital requirements can be effective in mitigating global warming and the associated climate-related physical risks. Thomä and Gibhardt (2019) estimate the potential impact of a GSF and compare it with that of a DPF, focusing on factors ranging from 15% to 25%. They find that a GSF would have a limited effect on overall capital requirements, namely savings of about EUR 3–8 billion, depending on the breadth of the definition of green assets. Additional capital charges on brown assets would result in a EUR 14–22 billion higher bank capitalization, depending on the definition of brown assets. While a DPF could potentially reduce lending to environmentally harmful activities by up to 8%, the reduction in the cost of capital for green projects would probably be insufficient to make a difference. Dunz et al. (2021) assess the effectiveness of the introduction of a GSF and a carbon tax as alternative policy levers to support the greening of the economy. They find that a GSF could contribute to scaling up green investments only in the short term and potentially increase the risks to financial stability.

Lastly, Diluiso et al. (2021) argue that the integration of climate-related risks into the financial regulatory framework may lead to very different outcomes depending on the scheme implemented. In particular, in the case of a negative shock originating in the fossil fuel sector, capital requirements penalizing fossil fuels would significantly reduce the severity of a financial crisis but also slow down the recovery.

This paper contributes to this debate by assessing the potential impact of climate transition risk on EU banks’ balance sheets and establishing a basis for calibrating relevant prudential instruments.

We show how even a very small depreciation in such assets could trigger uncontrolled market dynamics, ultimately leading to significant losses for the banking system as a whole. We also show that the risks are concentrated in some countries. Lastly, carry out an extensive uncertainty analysis to explore under which conditions fire sale dynamics could be triggered. Based on these results, we propose a calibration for relevant capital add-ons.

The modelling approach includes several steps. First, the share of exposures to high-carbon activities is used to recalibrate the risk-weighted assets (RWA) of banks to reflect the increased riskiness of investing in harmful activities. For this, we test a whole range of values for the increased riskiness of high-carbon assets, from 0% to 30%. Second, the Systemic Model of Bank Originated Losses (SYMBOL; see De Lisa et al., 2011), that is, a micro-simulation model based on individual bank balance sheet data, is used to generate crisis scenarios and estimate the aggregated loss distribution for the banking sector under each calibration for the increased riskiness of high-carbon activities. We then consider two main scenarios, namely (i) the case of a financial crisis triggered by climate-unrelated factors, under a static balance sheet assumption, where transition risks are additional to the baseline, and (ii) the case of fire sale dynamics triggered by a small depreciation in high-carbon assets under a dynamic balance sheet assumption.

In the first scenario, we compare aggregate losses in the case of a crisis, comparable in magnitude to the global financial crisis, both assuming no transition risk and accounting for transition risk. We calculate that, in this case, the extra losses due to transition risk are small compared with overall losses, although not irrelevant, unless high-carbon assets are assumed to be much riskier than comparable assets. In other words, in the case of a systemic crisis due to for example, a recession, the main driver of losses would be the recession, not transition risk. However, we show that transition risks are concentrated in a few countries, and, in those countries that are particularly exposed, losses could substantially increase.

In the second scenario, to model possible second-round effects, we start from the same simulation results and check how many banks would fail due only to their particularly high exposure to transition risk in a business-as-usual setting. We then investigate under which conditions these bank defaults could trigger a fire sale mechanism involving high-carbon assets, starting with an initially contained depreciation of these assets. The initial shock is amplified via second-round effects, and it eventually reaches a systemic scale as further depreciations of high-carbon assets put under stress an increasingly larger number of banks, despite portfolio reallocations triggered by market developments. These dynamics stop only when a new equilibrium is reached, which happens, based on our results, after losing around 0.7–0.9% of total assets, that is, more than EUR 400 billion, considering the size of the EU banking sector. It should be noted that this scenario does not necessarily correspond to a disorderly transition in the Network for Greening the Financial System narrative (see, for example, Network for Greening the Financial System, 2020, Network for Greening the Financial System, 2021), as market dynamics move much faster than economic transitions. In other words, a fire sale such as the one we model could unfold in the very short term, as it would relate much more to investors’ **expectations** of the green transition than to the actual progress of the transition. Given its short-to medium-term focus, our model can be used as a climate stress-testing tool.

Lastly, we investigate under which conditions the introduction of additional capital requirements, accounting for transition risks faced by each bank, could effectively protect the system (see Section 2). We propose a calibration for such a measure that would ensure that a fire sale does not even start. Virtually no bank would fail because of its transition risk exposure, thanks to the capital add-on, and hence no uncontrolled market dynamics would be triggered. We estimate that a capital add-on corresponding to 0.9% of RWA on average would be adequate. However, such a policy measure might be only temporary until the economy and, in turn, banks’ balance sheets become green enough.

The paper is organized as follows. Section 2 provides an overview of relevant policy initiatives. Section 3 explains how the inputs to the model were derived, notably estimates of augmented RWA accounting for transition risk. Section 4 provides an overview of the main modelling framework. Section 5 investigates the case of a systemic banking crisis not triggered by transition risk, while Section 6 models a crisis triggered by transition risk. Lastly, Section 7 provides the conclusions.

## 2 Policy background

Several policy initiatives have been introduced to raise awareness among financial institutions about the need to integrate climate-related risks in risk management processes, to make the regulatory and supervisory framework fit for purpose and to develop suitable monitoring and assessment tools. The ultimate goal is to capture these risks in supervisory and policy decision-making processes, limit the further build-up of climate-related financial risks and enhance the resilience of individual banks and the financial system as a whole. The policy framework is more advanced in relation to the monitoring and assessment of transition risks, but there is no doubt that physical risks also need to be addressed.

In its report on environmental, social and governance risk management and supervision, the European Banking Authority (2021) provides guidance on how banks and supervisors should integrate these risks. The Bank for International Settlements (2021b) explores how climate-related financial risks can affect the banking system, concluding that ’traditional risk categories used by financial institutions and reflected in the Basel Framework can be used to capture climate-related financial risks’.

In July 2021 the European Commission launched its renewed strategy for financing the transition to a sustainable economy (see European Commission, 2021a). Several of the main actions address the need for the financial system to identify and manage sustainability risks, including climate-related risks. In this context, a key issue relates to the ability of banks to absorb financial losses that may arise from exposure to companies and sectors negatively impacted by the transition. The strategy envisages potential amendments to banks’ capital requirements as a means to enhance economic and financial resilience to sustainability risks.

The European Commission’s proposal for a review of the capital requirements directive (see European Commission, 2021b), published a few months later, explicitly mentions the use of a systemic risk buffer to address risks related to climate change. Back in 2018, in the action plan on financing sustainable growth, the European Commission (2018) proposed a strategy to help channel financial investment towards green activities. One of the suggestions was to investigate the possibility of using banks’ capital requirements, the rationale being to align banks’ investment decisions with sustainable finance goals. The action plan refers to the introduction of a GSF to reduce capital requirements for green investments, provided that they can safely be regarded as less risky than other investments.^1^ The European Banking Federation (2017) is also in favour of a GSF for certain assets classified as sustainable under the EU taxonomy.

This idea, however, has provoked a range of unfavourable reactions. It has been challenged mainly with the argument that **the extra risk of brown assets does not make green assets extra safe** (see Boot and Schoenmaker, 2018). Opponents of the GSF also claim that such a discount in capital charges reduces the buffers needed by banks to withstand future losses (see Dankert et al., 2017).

Essentially, supervisors argue that under no circumstances should the need to make the financial sector contribute to the green transition lead to an unjustified loosening of capital requirements, which were increased after the global financial crisis to secure the resilience of banks and ensure financial stability. On the contrary, the introduction of a DPF for fossil fuel-intensive assets, reflecting the systematic and potentially systemic risk of brown assets, has been attracting growing attention. This alternative would not only avoid the depletion of capital buffers, including those needed to withstand additional losses linked to physical risks, but also discourage further investments in environmentally harmful activities, thereby contributing to favouring a shift towards green investments (see Grantham Research Institute on Climate Change and the Environment, 2017). Moreover, if transition risks are material for certain asset categories and not fully incorporated, a DPF would also increase financial stability.

Lastly, it should be noted that any suitable prudential capital buffer, be it a dedicated penalizing factor for high-carbon exposures or the systemic capital buffer itself, could potentially be used to protect the system, as long as it reflects the systemic nature of the risk. As concluded in a recent (European Banking Authority, 2022) discussion paper, the Pillar 1 framework already includes tools that allow the inclusion of sustainability-related financial risks, including climate-related risks. In this respect, the results in this paper are relevant from a prudential perspective; however, they can also be used in the context of Pillar 2 measures.

## 3 Data and other model inputs

**Fig. 1:**
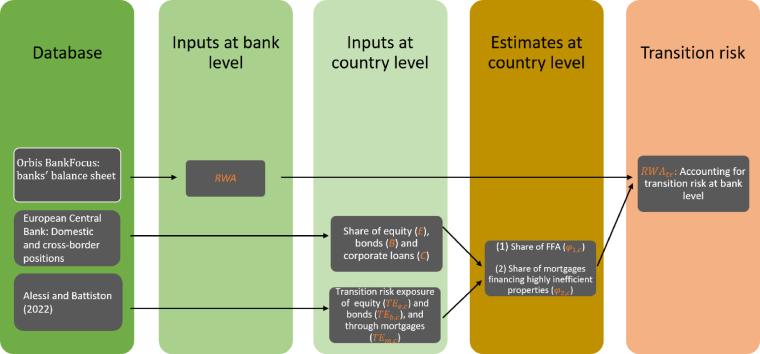
Summary of inputs for the estimation of transition risk exposures on banks’ balance sheets.

We used several types of input to estimate banks’ exposure to transition risk, which is the starting point of the modelling framework. As shown in Fig. 1, we used bank-level balance sheet data, as well as aggregate statistics at the country level, and country-level estimates of various quantities for which bank-level information was not available.

Looking at the column ‘Database’ in Fig. 1, individual banks’ balance sheet data, consolidated at the level of the banking group, were sourced from Orbis BankFocus. In particular, we used total assets (TA), RWA and total regulatory capital (K).^2^ The final dataset covers 461 EU banks located in the EU-27, including commercial and cooperative banks, savings institutions, and real estate and mortgage banks. They account for around 89% of the TA in the EU banking system. The data refer to 2020.

Together with balance sheet data, a key input to the model was the size of high-carbon and fossil fuel-related assets in banks’ balance sheets, the share of which was used to recalibrate banks’ RWA to reflect the increased riskiness of financing harmful activities. In particular, we adjusted the calculation of RWA by assuming that securities and corporate loans that finance high-carbon and fossil fuel-related activities (FFAs), as well as mortgages financing particularly energy-inefficient buildings, could be riskier than other assets.

Looking at the column ‘Inputs at bank level’ in Fig. 1, the augmented RWA accounting for transition risk ($RWA_{tr}$) were derived for each bank as follows:

(1) $$RWA_{tr}=\beta \left(\phi_{1,c}+\phi_{2,c}\right)RWA+\left(1-\phi_{1,c}-\phi_{2,c}\right)RWA,$$

where β is the parameter representing the extra riskiness of fossil fuel-related assets, ($\phi_{1,c}$) represents the share of FFAs in banks’ balance sheets and ($\phi_{2,c}$) represents the share of mortgages financing highly energy-inefficient properties. The results in Sections 5.2, 6.3 were obtained by investigating values for β between 0% and 30%.

Estimates of the ($\phi_{i,c}$) shares were obtained at the country level, starting from estimates based on the methodology presented in Alessi and Battiston (2022). This approach takes the EU taxonomy for sustainable activities as a reference for the identification of harmful activities, as the do no significant harm (DNSH) technical screening criteria provided by the taxonomy *de facto* defines harmful activities. The DNSH criteria are complemented by the climate policy relevant sectors classification (CPRS) to cover those sectors of the economy that are not included in the taxonomy, for example fossil fuels. Based on these classifications, the authors developed standardized transition risk exposure coefficients for all sectors of the economy. These were used to estimate the exposure to transition risk of European investors, based on confidential security-by-security data from the European Central Bank. These estimates also include the exposure to transition risk of banking sectors in the various countries. These confidential figures cover, in particular, estimates of the transition risk exposure of national banking systems through equities ($TE_{e,c}$), bonds ($TE_{b,c}$) and mortgages ($TE_{m,c}$), including cross-border balance sheet items. While we acknowledge that other definitions and classifications may be used to identify high-carbon activities, other approaches available in the literature require granular exposure-by-exposure data, in which individual assets are assessed based on the characteristics of the issuer or borrower.

Next, using breakdowns of domestic and cross-border positions within the euro area provided by the European Central Bank,^3^ we derived the share of equity (E), bonds (B), mortgages (M) and corporate loans (C) in banks’ balance sheets at the country level (column ‘Inputs at country level’ in Fig. 1), to which we applied the coefficients above to obtain the share of high-carbon assets at the country level (the column ‘Estimates at country level’ in Fig. 1).^4^

(2) $$\phi_{1,c}=TE_{e,c}\cdot E_{c}+TE_{b,c}\cdot \left(B_{c}+C_{c}\right),$$

(3) $$\phi_{2,c}=0.7\cdot M_{c}.$$

**Fig. 2:**
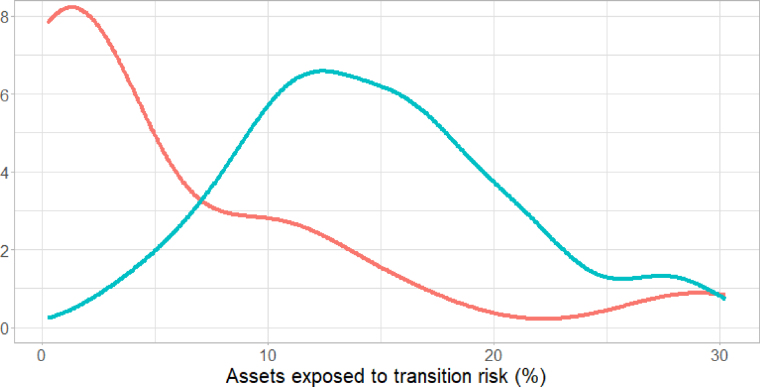
Share of assets exposed to transition risk: red line refers to FFA-related assets and the blue line refers to mortgages for energy-inefficient buildings.

Fig. 2 shows how the share of assets exposed to transition risk (on the *x*-axis) varies across countries, considering all high-carbon assets (in red) and excluding mortgages (in blue). The distribution densities show that, in most cases, the exposure to transition risk is lower than 2% of banks’ TA. This increases to 14% on average when mortgages are included, showing that mortgages play a key role in shaping the average exposure to transition risk.

**Fig. 3:**
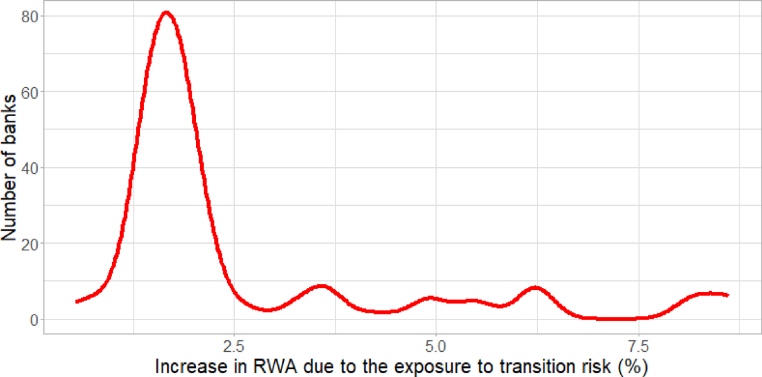
Distribution of increases in RWA due to exposure to transition risk.

Fig. 3 illustrates the impact of accounting for transition risk on the size of RWA for the sample. The *x*-axis shows the estimated increase in RWA, while the *y*-axis reports the number of banks. The graph shows that, when accounting for transition risk, with β=13%, the range of the increase in RWA is between 1% and 2% for the majority of banks. However, the distribution has a very long right-hand tail, as a small fraction of banks may face larger increases in their RWA, even exceeding 7.5%.

Lastly, to account for estimation uncertainty around ($TE_{e,c},\;TE_{b,c}$) shares, we generated quasi-random numbers for these estimates by considering their distribution across countries, and we made each country-specific estimate vary within 1 standard deviation. These values were inputs to the model described in the following sections. We also checked how sensitive model results were to this particular input. The results in Appendix B indicate that they did not have a significant effect on the model output.

## 4 Simulation model

The modelling strategy was based on SYMBOL (see De Lisa et al., 2011). The model, based on bank level data, essentially simulates banking crisis scenarios where individual banks may default based on their actual capital (K) and on the probability of default (PD) attached to their portfolio.

The bank’s portfolio PD was derived by inverting the Basel formula, which expresses minimum capital requirements as a function of the PD of a bank’s portfolio and its RWA:

(4) $$CR\left(PD\right)=LGD\cdot N\left[\frac{\sqrt{R}N^{-1}\left(\frac{0.08\cdot RWA}{TA}\right)+N^{-1}\left(PD\right)}{\sqrt{1-R}}\right]-PD,$$

where $R_{i}$ is the correlation among exposures in the portfolio, LGD is the loss given default, equal to 0.45 as per Basel II regulation,^5^ N is the normal distribution function and $N^{-1}\left(\alpha_{ij}\right)$ is correlated normal random shocks.

Bank-specific PDs were then used to generate losses for individual banks via a Monte Carlo simulation, where randomness was introduced by sampling the underlying shocks. The output of the Monte Carlo simulation was an I×J matrix of unexpected losses ${\left(\text{GL}\right)}_{ij}$, obtained as follows:

(5) $${GL}_{ij}=LGD\cdot N\left[\sqrt[{}]{\frac{1}{1-R_{i}}}N^{-1}\left(PD_{i}\right)+\sqrt[{}]{\frac{R_{i}}{1-R_{i}}}N^{-1}\left(\alpha_{ij}\right)\right]-EL_{i},$$

where the first term generates the total amount of losses and the second one, ($EL_{i}$), approximates the expected losses.^6^ Specifically, i=1,…,I refers to the banks in the sample, and j=1,…,10000 denotes the model iteration. The $\alpha_{ij}$ shocks are correlated, as they are defined as the sum of a common shock, $Z_{j}$, and a bank-specific shock, $W_{ij}$, as follows:

(6) $$N^{-1}\left(\alpha_{ij}\right)=l\cdot Z_{j}+\sqrt[{}]{1-l^{2}}\cdot W_{ij},$$

where l are the loadings, $W_{ij}$ are the idiosyncratic shocks, and $Z_{j}$ is a common shock that might be linked, for example, to overall economic development. The standard version of the model, which is used in this paper, sets l to yield a fixed correlation of 0.5 across the $\alpha_{ij}$.^7^ The shocks $Z_{j}$ and $W_{ij}$ are drawn from a standardized normal distribution.

While expected losses should be covered on an ongoing basis by provisions and write-offs, unexpected losses relate to potentially large losses that occur rather seldom and therefore should be covered by capital. Failure of any individual bank is determined by the size of the unexpected losses compared with the actual regulatory capital available to absorb them. In other words, banks fail when simulated unexpected losses exceed the total actual capital^8^:

(7) $$Failure_{ij}:{GL}_{ij}>K_{i}.$$

Some of the model iterations, most likely those in which the sampled shock is larger, will result in at least one bank defaulting. In these cases, the following two components are summed for each bank: (i) losses that cannot be absorbed by capital; and (ii) recapitalizations needed to bring the bank back to a viable status, that is, a regulatory Tier 1 capital ratio of 4.5% of RWA plus systemic Pillar 1 buffers (SRB), for example the buffers for global systemically important institutions and other systemically important institutions.^9^ From here on, we then define the **losses** for each bank and each iteration as the unexpected losses in excess of capital plus recapitalization needs ($ExLR_{ji}$):

(8) $$ExLR_{ji}=\max\left(GL_{ji}-K_{i}+4.5\text{\%}RWA_{i}+SRB_{i},0\right).$$

Excess losses plus recapitalization needs for individual banks yield the following aggregate loss distribution ($L_{i}$) for each iteration:

(9) $$L_{j}=\overset{I}{\underset{i=1}{\sum }}ExLR_{ij}.$$

Depending on the banks in the scope of the analysis, the aggregate distribution can be at the country or EU level. As with individual bank losses, each point in the distribution (i.e. each iteration) is associated with a different level of distress.

This modelling framework can accommodate different degrees of commonality by allowing for various shock correlation structures, while a contagion mechanism is not explicitly considered. We do not explicitly model contagion effects through the interbank market for three main reasons. First, contagion would introduce an additional layer of complexity, increasing the uncertainty around the results. Second, modelling contagion dynamics is quite challenging, as public data are not fit for the purpose of calibrating the network. Third, the comprehensive crisis management and deposit insurance framework put forward by the Commission after the last financial crisis (notably, the bail-in tools and the establishment of resolution funds) is expected to prevent the spreading of contagion, as distressed banks would be resolved, liquidated or recapitalized well before direct contagion effects could materialize (see, for example, Benczur et al., 2017).

**Fig. 4:**
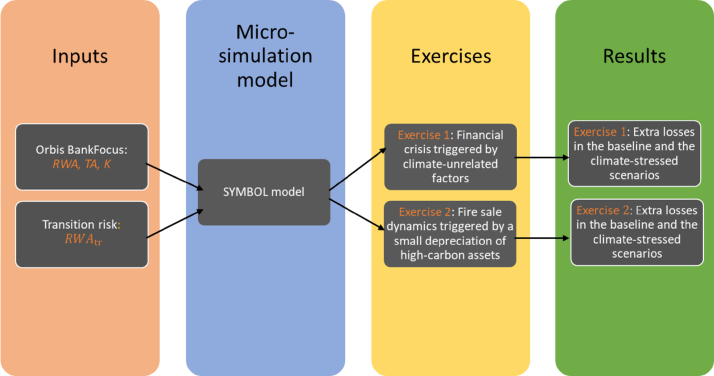
Description of the empirical analysis.

We used the SYMBOL model as the basis for two complementary exercises, which we describe in detail in the following sections. As summarized in Fig. 4, in the first exercise we took as a baseline a stressed macroeconomic scenario, corresponding to the SYMBOL iterations associated with the largest shocks and the largest bank losses. Against this baseline, we investigated the impact of the additional materialization of transition risk. In a second exercise, we instead considered SYMBOL iterations where no banks default and used this business-as-usual, benign macroeconomic scenario as a baseline. Similarly to the previous exercise, we investigated the impact of the materialization of transition risk against this baseline.

## 5 A crisis not triggered by transition risk

### 5.1 Modelling framework

In this section, we used the model presented in Section 4 to assess the impact of transition risk in the context of a systemic banking crisis that is **not** triggered by climate transition risk. We did so by comparing the following two situations.

1. Baseline scenario. The macroeconomic scenario is stressed; however, transition risk is absent (i.e. current RWA properly reflect the level of risk).
2. Stressed scenario. On top of macroeconomic stress, transition risk materializes. This is introduced by adjusting RWA upwards for exposures to high-carbon and fossil fuel-related assets, while keeping regulatory capital constant (see Fig. 3).

We focused on the (far) right-hand tail of the loss distribution, which is associated with a severe, but plausible, banking crisis due to a severe recession. Technically, this part of the distribution corresponds to values of $Z_{j}$, which are farther than 3 standard deviations from the mean.^10^ This corresponds to the following iterations $\overset{˜}{j}$:

(10) $$\overset{˜}{j}=\left\{j\;\text{such that}\;Z_{j}>E\left(Z_{j}\right)+3\text{std}\left(Z_{j}\right)\right\}.$$

Systemic losses (SL) were then computed by taking the expected value of aggregate bank losses (as defined in Eq. (9)) across the selected iterations, which corresponds to the expected shortfall concept:

(11) $$SL=E_{j}\left[{\left(L\right)}_{j}|j\text{in}\overset{˜}{j}\right].$$

By comparing systemic losses with and without transition risk one can derive how much additional capital banks should hold to provide an adequate level of protection in the face of a crisis should transition risks materialize.

### 5.2 Results

In a crisis situation not triggered by transition-related factors, the contribution of additional transition risks to overall losses is relatively modest in absolute value but not irrelevant. While the exact figure depends on the underlying riskiness of high-carbon assets, for any moderate value of β aggregate bank losses for the EU-27 increase only relatively modestly when considering transition risk. For β=13%, the benchmark value used in Section 6, aggregate losses increase by around 10% on average. Only for larger values of β, close to 30%, do aggregate losses increase by 20%. Our interpretation is that, in the case of a systemic crisis due to an economic shock, the key shock is this one, not the materialization of transition risk. In fact, losses due to transition risks are proportionally greater in less severe systemic crises. For example, in the case of a shock of 1 standard deviation (as opposed to 3 standard deviations, which is the benchmark case), losses would increase by 25% if β=30%.

**Fig. 5:**
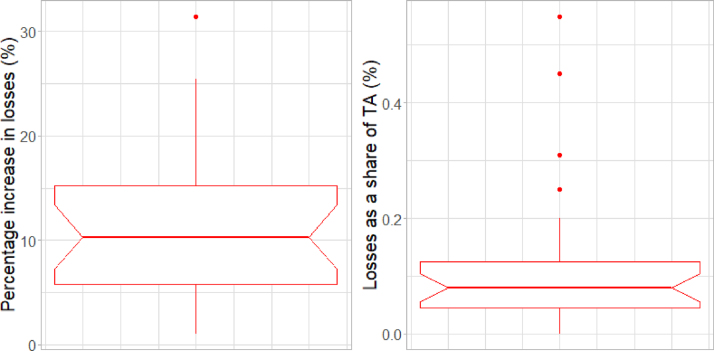
Distribution across countries of loss increases in percentage terms (left panel) and of losses as a share of TA (right panel) for β=13%.

Although transition risks seem to bring about contained additional financial losses, our results show how a few countries could be affected to a greater degree. Fig. 5 shows the distribution of loss increases across countries, for β=13%. The situation is quite heterogeneous across jurisdictions, with some countries subject to very mild (or almost zero) impacts, while in others losses can increase substantially with respect to the baseline of no transition risk. This is the case for five countries in particular, where the increases in overall losses are more than 15%. For more extreme values of β, overall losses can increase by up to 70%.

## 6 Transition risk as a trigger of a crisis

### 6.1 Modelling framework

In this second application of the model, we investigated under which conditions transition risk could be the trigger of a systemic crisis even in the absence of a large shock to the common economic factor. In this exercise, the two scenarios were defined as follows.

1. Baseline scenario. The macroeconomic scenario is benign (business as usual) and transition risk is absent.
2. Stressed scenario. The macroeconomic scenario remains benign but transition risk materializes.

We started by identifying those banks that would fail because of transition risk materialized (stressed scenario) but would not fail otherwise (baseline scenario). We then modelled a mechanism whereby, owing to these initial defaults, high-carbon and fossil fuel-related assets are sold off. A depreciation of these assets, in turn, puts more banks under stress, leading to further sell-offs. The fire sale continues until the system reaches a new equilibrium, that is, no more banks default.

Formally, the banks that default only in the stressed scenario satisfy the following condition:

(12) $$\left\{\begin{array}{cc} ExLR_{ij}\; & =0\;\text{without transition risk (baseline)},\\ ExLR_{ij}\; & >0\;\text{with transition risk}. \end{array}\right)$$

The systemic loss due to transition risk is computed considering only those banks. This is a first-order systemic loss, as so far no amplification mechanisms have been considered.

In the second step, we introduced dynamic features into the model, namely: (i) a dynamic balance sheet, that is, the possibility of banks being able to reallocate their portfolios; and (ii) a depreciation of FFAs (see Section 3). In particular, we assumed that bank failures may trigger a sequence of fire sales of FFAs, which is reflected on the one hand in a lower share of FFAs on banks’ balance sheets and on the other hand in lower market prices for those assets.^11^

The fire sale mechanism was modelled as follows. We assumed that the initial sell-off of assets exposed to transition risk would lead to only a very limited depreciation of such assets, corresponding to $\delta_{t_{1}}$. The size of the depreciation at each round of the fire sale was modelled by linking the depreciation size to the number of banks defaulting. It is reasonable to assume a sharper depreciation if the number of banks defaulting at each round increases, and a more gradual depreciation when bank defaults start to decrease. Formally, at each round of the fire sale ($t_{r}$) the size of the depreciation is proportional to the number of failing banks in the previous round:

(13) $$\delta_{t_{r}}=\max\left(\frac{D_{t_{r}-1}-D_{t_{r}-2}}{D_{t_{r}-2}},\delta_{t_{1}}\right)\text{if}t_{r}>1\text{and}D_{t_{r}-1}>0,$$

where D is the number of defaults. The sequence stops when no more banks fail.

Lastly, while triggering further defaults among banks in the system, the fire sale also implies changes in banks’ balance sheets, as the share of FFAs decreases. Formally, we assumed that when the fire sale starts, together with a depreciation of FFAs, their share in banks’ balance sheets also decreases by a percentage, θ:

(14) $$FFA_{t_{r}}=\left\{\begin{array}{cc} \; & FFA_{t_{r}}\cdot \left(1-\theta \right)\;\text{if}\;t_{r}=0\\ \; & FFA_{t_{r}-1}\cdot \left(1-\delta_{t_{r}}\right)\text{if}t_{r}>0. \end{array}\right)$$

With respect to subsequent periods, it becomes increasingly difficult to sell assets that are subject to a fire sale; hence, we assume for simplicity that the share of FFAs did not significantly change. However, it would be possible to use any suitable functional form.

### 6.2 Model calibration

The modelling framework described above entails five key parameters, namely β, the increased riskiness of high-carbon assets, δ, the size of the initial depreciation of high-carbon assets, θ, the size of the initial sell-off of high-carbon assets, and the shares of high-carbon bonds and equities, $TE_{b,c}$ and $TE_{e,c}$, respectively.

In order to calibrate them, we carried out an uncertainty analysis, running the model 64 times, with the parameters varying as follows: β=[0%,30%], δ=[0%,2%] and θ=[0%,20%], while $TE_{b,c}$ and $TE_{e,c}$ were generated using quasi-random numbers within 1 standard deviation.

**Fig. 6:**
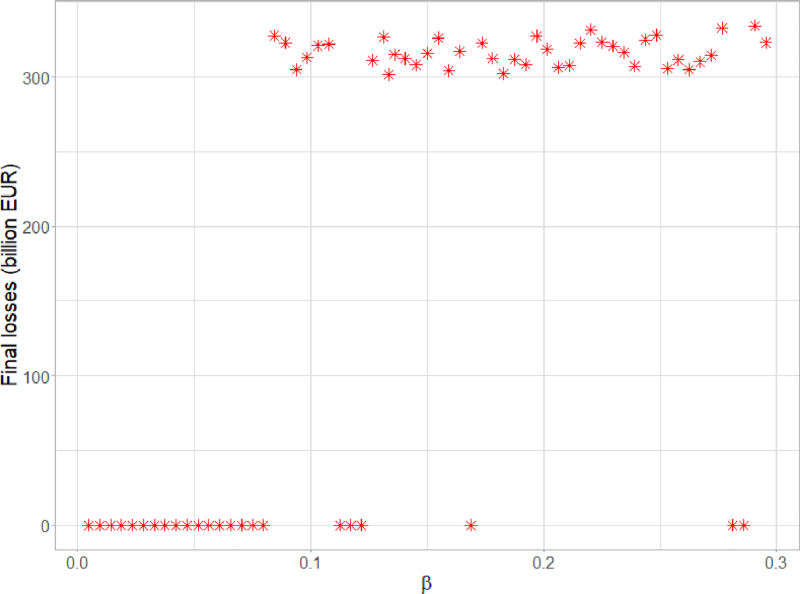
Calibration of β, representing increased riskiness of high-carbon assets; the graph shows the size of losses under each value of β.

Fig. 6 shows the final amount of bank losses yielded by the model for various levels of β, generated using quasi-random numbers in the [0%, 30%] interval. For β<8% the level of aggregate losses is most of the time zero or very small, indicating that a level of increased riskiness of high-carbon assets of up to 8% in this setting would not pose a systemic threat. For β>8% the fire sale could already be triggered in some simulations, yielding a large amount of losses. However, this does not happen consistently in all simulations until β reaches 13%. For β>13% the amount of final losses levels off, with virtually all simulations yielding a large amount of losses. Based on these results, we took a conservative stand and set β at 13% in the benchmark analysis.

**Fig. 7:**
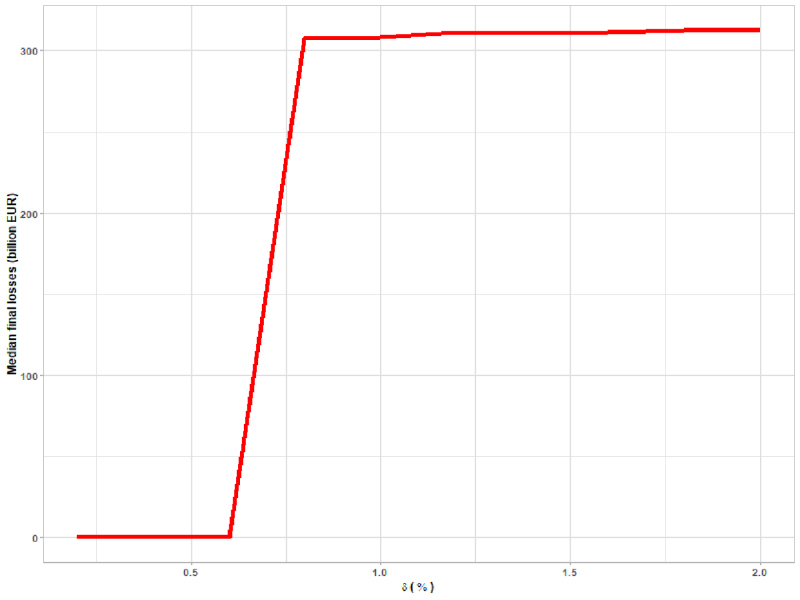
Calibration of δ, representing the initial depreciation; the graph shows the size of median losses under each value of δ.

The same approach was used to calibrate δ, the initial depreciation. We fixed the other parameters and let δ vary between 0% and 2%, with a grid step of 0.2 percentage points. As shown in Fig. 7, a level of depreciation corresponding to 0.8% is enough to generate additional bank defaults and potentially trigger a fire sale. At the same time, the overall results of the modelling exercise in terms of aggregate losses did not change for larger values of δ. Hence, we set δ at 0.8%.

With respect to θ, one should consider that the higher the initial sell-off, the smaller the final amount of bank losses. Indeed, the more assets that banks are able to shed at the very beginning of the process, the lower the losses they will incur at the end. At the same time, a too large value for the initial sell-off, as well as being unrealistic, will make a fire sale impossible, as a large part of the assets is sold at $t_{0}$. We calculated that a fire sale cannot start at values of θ larger than 20%. In fact, it would be difficult to defend a parameterization corresponding to banks being able to shed an even larger portion of their FFAs, in a context where all financial market participants are trying to do the same. Additionally, for θ larger than 16% aggregate losses level off; hence, we used 16% as an upper bound for θ. However, in contrast to β and δ, in the case of θ, values below 16% would also be reasonable. Hence, we ran the benchmark analysis for θ ranging between 0%, that is, a static balance sheet assumption, and 16%.

Lastly, the model output did not show significant effects due to uncertainty in $TE_{b,c}$ and $TE_{e,c}$.

### 6.3 Results

Based on the SYMBOL simulation and considering an increased riskiness of high-carbon assets of 13% (see above), in a business-as-usual (i.e. no crisis) scenario, around 1 out of 8 of banks could fail should transition risks materialize, with one or two actually failing in each Monte Carlo iteration. Furthermore, should a 0.8% depreciation of such assets follow (see above), this would be enough to trigger additional bank defaults. In addition, some other particularly exposed financial institutions would suffer substantial second-round losses, due to the depreciation of high-carbon assets.

**Fig. 8:**
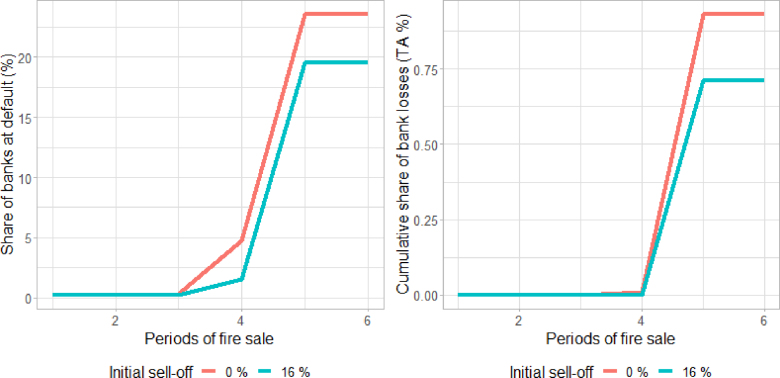
Evolution of the fire sale in terms of cumulative share of banks at default (left panel) and cumulative share of bank losses as a share of TA (right panel), under different levels of sell-off (light blue sell-off is equal to 0%; dark blue sell-off is equal to 16%).

Fig. 8 (left panel) shows the cumulative share of banks at risk over the total number of banks in the sample at each period of the fire sale and corresponding to levels of initial sell-off between 0% and 16% (see above). As shown in the right panel, the fire sale stops only after around 20%–24% of banks fail.^12^ In reality, this would not happen, as bank recovery mechanisms and troubled assets purchase programmes would be triggered long before such a large number of banks became insolvent. Therefore, these results show that, should such a crisis arise, we should be prepared to use public finances to avoid systemic consequences of this magnitude.

Looking at aggregate financial losses, Fig. 8 (right panel) shows their evolution as a percentage of TA under initial levels of sell-off corresponding to 0% and 16%. The numbers suggest that, given the small size of the banks defaulting in the first phases of the fire sale, the losses for the banking system as a whole would initially be very contained. However, the dynamics would then become exponential. We calculated that aggregate losses would initially remain under EUR 1 billion for some rounds but then start to sharply increase under all paths. Up to this point, implementing a backstop could still manage the crisis with limited recourse to public finances. Going forward, the extra risk of holding harmful assets under a fire sale dynamic could lead to average losses corresponding to 0.7–0.9% of TA (with a standard deviation of 0.02%),^13^ that is, around EUR 400 billion for the EU banking sector as a whole.

**Fig. 9:**
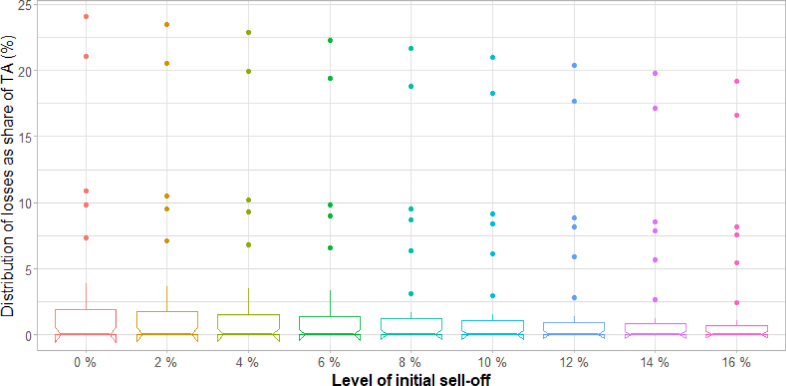
Distribution across banks of losses as a share of TA, after a fire sale and under different levels of initial sell-off.

As shown in Fig. 9, the distribution of additional losses at the bank level for all levels of initial sell-off considered is highly skewed to the left, with a relatively limited number of cases suffering disproportionately large losses. The risk is thus not only potentially large but also highly unpredictable in nature, owing to the large variance of the loss distribution. Furthermore, transition risks are concentrated in this case.^14^

**Fig. 10:**
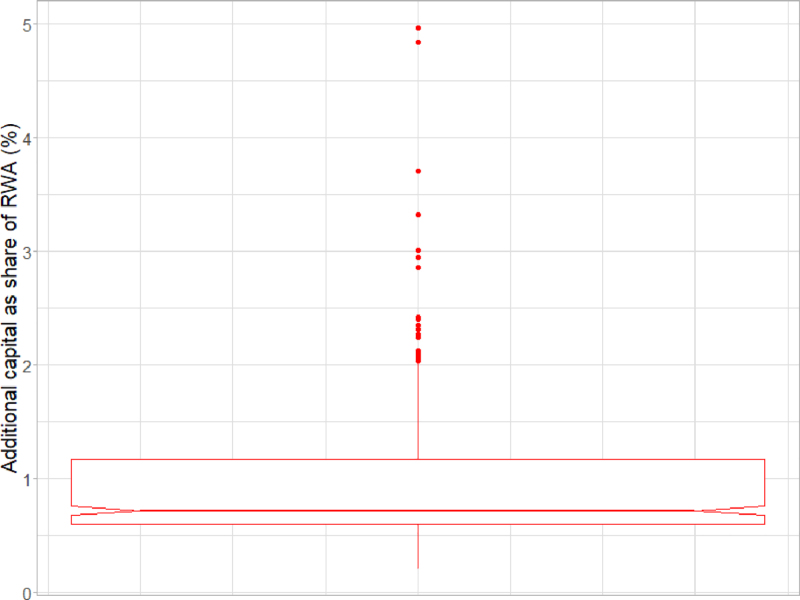
Distribution across banks of additional capital needed to offset transition risk, as a share of RWAs.

An extra capital add-on proportional to the transition risk faced by each institution could succeed in protecting the system. If all banks were adequately protected, no or very limited additional defaults due to the realization of transition risks would be observed outside the baseline, and a fire sale would not even start. Based on these results, we calculated that a capital add-on of around 0.9% of RWA on average, in absolute terms, or 5% of existing capital in relative terms, would be sufficient. However, Fig. 10 shows that banks where transition risks are concentrated would need to set aside more capital, up to 5% of RWA in very extreme cases.

To sum up, Table 1 reports total bank losses in the various scenarios considered. In case 1, the baseline scenario is characterized by macroeconomic stress, while in case 2 this is absent. The stressed scenario in both cases consists of considering the materialization of transition risk against the baseline. Estimates under the stressed scenarios correspond to β between 13% and 30%.

## 7 Conclusions

In this paper we propose a methodology for assessing the potential bank losses associated with transition risks in the short run. Considering a stressed baseline macroeconomic scenario, we show that transition risks would increase overall losses only modestly — unless high-carbon assets are much more risky than currently considered. However, banks in some countries would suffer much higher losses than in others.

Moreover, we calculate that, even in a business-as-usual scenario, a few banks could default if a large enough transition risk materializes. We identify the conditions under which, should these defaults trigger disorderly market adjustments, subsequent fire sale dynamics could lead to significant losses for the EU banking system as a whole and to the default or distress of a large number of institutions.

**Table 1:** Total bank losses in the various scenarios considered (as a share of TA).

	Case 1 -	Case 2 -
	macro stress	climate trigger
Baseline scenario	0.07%	0%
Stressed scenario	0.08%	0.7–0.9%

This fire sale process can be effectively tackled with the introduction of a bank-specific capital add-on accounting for transition risks in banks’ balance sheets, which, based on current exposures, should be around 0.9% of RWA on average. However, as the economy becomes greener and banks’ balance sheets become less exposed to high-carbon assets, this capital buffer could be reduced as the risk of a fire sale and the associated potential losses become lower.

**Table 2:** Sample descriptive statistics.

	Banks (n)	TA (billion EUR)	RWA (billion EUR)	K (billion EUR)
AT	26	651	290	56
BE	14	1,138	388	78
BG	9	48	25	6
CY	9	48	22	4
CZ	12	247	91	21
DE	19	5,888	2,036	357
DK	22	1,095	259	61
EE	7	40	16	5
GR	6	293	167	25
ES	44	3,883	1,496	248
FI	20	861	265	55
FR	100	12,550	3,120	588
HR	7	65	36	8
HU	14	143	77	15
IE	8	575	233	46
IT	43	3,119	1,102	207
LT	4	28	9	2
LU	8	218	81	17
LV	9	20	8	2
MT	4	23	9	2
NL	14	2,169	679	148
PL	14	361	208	39
PT	14	366	176	39
RO	8	76	38	9
SE	14	994	257	58
SI	7	40	24	4
SK	5	71	39	6

Overall, our findings support the idea that a materialization of transition risks could lead to the realization of a systemic event and possibly merit public intervention. Banks that are financing high-carbon activities could therefore be asked to increase their protection against the risks they could face owing to the low-carbon transition, as, under some scenarios, these losses could be sizeable and spill over into the wider banking sector.

Further research could investigate in more detail the impact of transition risk-related financial crises on public finances, as policymakers and academics are devoting increasing attention to the role of climate-related risk in debt sustainability. Looking at the use of this and similar models for stress-testing, a promising avenue for further research could be the modelling of different financial amplification mechanisms and second-round effects. Lastly, the modelling framework presented in this paper could be used to capture some dimensions of the ‘green bubble’ narrative, by investigating the implications of assuming that green assets are riskier than similar assets. Given the currently limited share of green assets on banks’ balance sheets, sizeable losses are likely to occur only under very extreme parameterizations.

## CRediT authorship contribution statement

**Lucia Alessi:** Writing – review & editing, Writing – original draft, Conceptualization. **Erica Francesca Di Girolamo:** Writing – review & editing, Writing – original draft, Supervision, Project administration, Formal analysis, Conceptualization. **Andrea Pagano:** Writing – review & editing, Writing – original draft, Visualization, Validation, Software, Formal analysis, Data curation, Conceptualization. **Marco Petracco Giudici:** Writing – review & editing, Methodology, Conceptualization.

## Figures and Tables

**Fig. 11:**
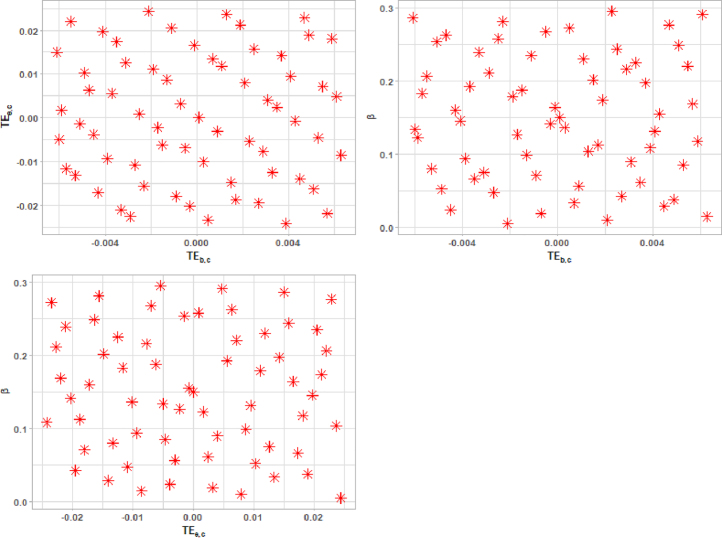
Parameters’ space for bonds, equity and penalizing factor.

**Fig. 12:**
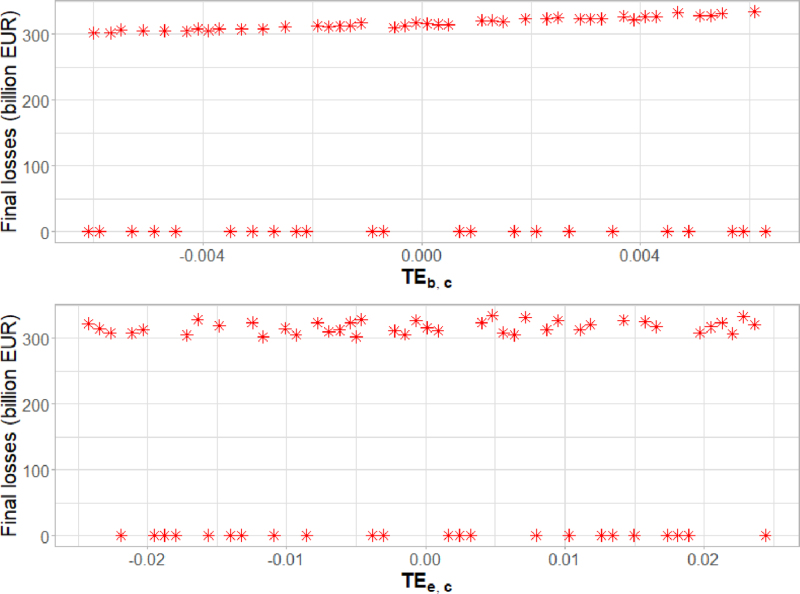
Effect of bond and equity uncertainty on the model output.

## Appendix A Data inputs

See Table 2.

## Appendix B Uncertainty analysis of the penalizing factor, share of brown bonds and equity

We used quasi-random numbers (i.e. Sobol sequences; see Saltelli et al., 2004) to assess the uncertainty in the model output due to the uncertainty around the calibration of the key β parameter, that is, the increased riskiness of high-carbon assets, and the parameters representing the shares of bonds and equities financing high-carbon activities.

We constrained the uncertainty for bonds and equities based on the variability of the country estimate, that is, we let these parameters vary within 1 standard deviation. As for β, it varied in the range [0,0.3]. Fig. 11 shows these three parameters’ space.

Section 6.2 discusses the importance of the β parameter, showing in Fig. 6 how the model output, that is, the final losses due to the fire sale, is sensitive to its values. In contract, the other two parameters do not show any significant effects on the model output. As shown in both panels of Fig. 12, the level of final losses (*y*-axis) is independent of the value of the parameters (*x*-axis).

## Footnotes

1 For more information, see the speech by European Commission Vice-President for the Euro and Social Dialogue, Financial Services Valdis Dombrovskis (https://ec.europa.eu/commission/presscorner/detail/en/SPEECH).

2 See Table 2 in Appendix A for descriptive statistics on the representativeness of the sample by country.

3 European Central Bank Data: Series 4. Domestic and cross-border positions of euro area monetary financial institutions (excluding the Eurosystem) and 2.2.1 Loans to households and non-financial corporations (EUR billions; not seasonally adjusted; outstanding amounts at end of period).

4 We also used $TE_{b,c}$ for corporate loans, as a specific exposure coefficient for loans was not available.

5 https://www.fdic.gov/analysis/cfr/working-papers/2006/2006-10.pdf.

6 Expected losses was calculated as the product of PD, LGD, and TA.

7 See Di Girolamo et al. (2017) for a discussion on a more sophisticated correlation structure.

8 See Benczur et al. (2017) for details.

9 The capital conservation buffer and other elements of the combined buffer were not included in the recapitalization requirements. Pillar 2 requirements were not included due to a lack of available data, especially for smaller banks. It should be noted that all capital was considered when absorbing losses, and that some of the buffers were simply not considered when calculating recapitalization needs following distress. This is a conservative choice in terms of the estimation of the final losses and recapitalization needs.

10 As $Z_{j}$ may be seen as a negative economic shock, this calibration is representative of a recession comparable to those observed during the global financial crisis and the COVID-19 pandemic.

11 We did not include mortgages, as such assets are not subject to mark-to-market accounting.

12 These banks account for 10% of sample TA.

13 The standard deviation was computed using the SYMBOL simulations involved in the fire sales.

14 Based on a set of regressions (available on request), losses at the bank level are explained, not surprisingly, by the share of high-carbon assets and mortgages for energy-inefficient buildings, as well as the usual controls.
